# Supplementary material for: When Mast Cells Run Amok: A Comprehensive Review and Case Study on Severe Neonatal Diffuse Cutaneous Mastocytosis
Source: Genes (Basel). 2023 Oct 29;14(11):2021. doi: 10.3390/genes14112021 (PMC10671269; doi:10.3390/genes14112021)
Supplement: Supplementary file 1 [file genes-14-02021-s001.zip › genes-2668075-supplementary.pdf]

	Case aggregation of DCM from the literature	Present case	Waters et al[12] 1957	Yasuda et al [13] 1963	Allison et al [14] 1967	Burgoon et al [15] 1968		Klaber et al [16] 1976	Harrison et al [17] 1979	Willemze et al [9] 1980	Oku et al [10] 1990
Number of cases	31	1	1	1	1	2		1	1	1	1
Sex (Male/Female)	19 M , 11 F, 1 NA	M	M	M	M	F	F	F	M	M	F
Darier sign (positive, negative or no information)	20 Positive, 1 Negative, 10 NA	Positive	NA	NA	Negative	Positive	Positive	Positive	NA	Positive	Positive
Systemic involvement (SI)	9 had SI, 14 without SI, 9 NA	Yes	Yes	No	Yes	Yes	Yes	No	Yes	No	No
Positive (elevated) blood and/or urine markers	15 had elevated markers, 7 had normal markers, 9 NA	Yes, elevated serum tryptase	NA	-	-	-	-	-	-	No	No
Bone-marrow biopsy (BMB)	7 had BMB with 4/7 with involvement, 13 without BMB, 11 NA	Yes - no involvement	NA	-	-	Yes - with involvement	Yes - with involvement	NA	NA	No	No
Biopsy	27 had a biopsy performed (skin the most common), 2 without biopsy, 2 NA	Yes - skin	NA	-	Yes - post-mortem	Yes - skin, skeletal muscle, lymph node, liver,	Yes - skin	Yes - skin	Yes - skin and liver	Yes - skin	Yes - skin
Special stains	12 had special stains, (Toluidine Blue and Giemsa most commonly used), 8 had no special stains , 11 NA	Toluidine Blue	NA	-	No	Giemsa, polychrome toluidine blue, Dominici, azure blue, colloidal iron, alcian blue, aldehydefuchsin, and PAS stains.	Giemsa, polychrome toluidine blue, Dominici, azure blue, colloidal iron, alcian blue, aldehydefuchsin, and PAS stains.	Thionin	NA	Toluidine Blue	Toluidine Blue
Ultrastructural analysis (UA)	2 had UA, 26 without UA, and 3 NA	No	No	No	No	No	No	No	No	Varying electon density of the granules dilated rough endoplasmic reticulum, little euchromatin, and a conspicuous nucleolus	Polygonal or spindle shaped, with numerous villous projections, homogeneous granules or electron dense, and pronounced extrusions of the granules into the extracellular matrix
IHC	5 had IHC for CD117, 3 had IHC for Tryptase, 9 without IHC, 14 NA	CD117 (c-kit) - diffuse positivity	-	-	-	-	-	-	-	-	-
Variant testing	KIT - p.(Ala502_Tyr503dup) - present case, 3 cases with KIT p.(Asp816Val), KIT dup A502Y503	*KIT* - c.1504_1509dup p.(Ala502_Tyr503dup)	-	-	-	-	-	-	-	-	-
Treatment	Main treatment was antihistamines and/or cromoglycate (23 cases) with systemic corticoids for systemic manifestations (8 cases), 1 case with Vitamin K and 1 case with Iron preperation, and 6 cases with NA	Cromoglycate	Vitamin K	Antihistamine	Tetracycline and systemic corticoids	Iron preparation	-	NA	Antihistamine	NA	Antihistamine
Follow-up	The longest follow-up was 27 years	2 years	5 years	3 months	8 days	8 years	20 months	6 months	4 years	27 years	8 months
Complications	Complications were reported in 17 cases with the most common spleno and/or hepatomegaly, 10 had none reported, 4 NA	Splenomegaly and cervical adenopathy	Mast cell leukemia	Shock	Systemic mastocytosis	Secondary bacterial infections	Possible gastrointestinal involvement	Possible coagulation abnormalities	NA	Large cutaneous tumors	NA
Vital status at follow-up (alive or dead)	24 were alive at follow-up, 6 were dead with 1 death of unknown cause	Alive	Dead	Dead	Dead	Alive	Alive	Alive	Alive	Alive	Alive

	Shah et al [4] 1998	Enomoto et al [18] 1999	Sethuraman et al [11] 1999	Hamaford et al [19] 2001	Fernandes et al [20] 2002	Mann et al [21] 2004	Walker et al [22] 2005	Duckworth et al [23] 2009	
Number of cases	1	1	1	1	1	1	1	2	
Sex (Male/Female)	M	M	M	NA	F	M	F	M	M
Darier sign (positive, negative or no information)	NA	Positive	Positive	NA	Positive	NA	NA	Positive	Positive
Systemic involvement (SI)	Yes	No	No	NA	NA	NA	NA	No	No
Positive (elevated) blood and/or urine markers	No	No	NA	NA	No	Yes - serum tryptase	Yes - serum tryptase, histamine , urine histamine and 1.4-methylimidazol acetic acid	Yes - serum histamine and elevated serum tryptase was reported but it is not clear which of the 2 reported cases had the increase	
Bone-marrow biopsy (BMB)	Yes - with involvement	No	No	NA	NA	NA	Yes - with involvement	No	No
Biopsy	Yes - skin and liver and post-morten skin and liver	Yes	Yes - skin	Yes - skin	No	Yes - skin	Yes - skin	Yes - skin	Yes - skin
Special stains	No	Toluidine Blue	No	NA	-	NA	Giemsa and naphthol ASD chloroacetate esterase	NA	NA
Ultrastructural analysis (UA)	No	No	No	No	-	No	No	No	No
IHC	No	No	No	No	-	NA	No	No	No
Variant testing	*KIT* Asp816Val	No	No	No	-	Yes - negative	Yes - negative, with one marker tested	No	No
Treatment	Antihistamine, cromoglycate, and systemic corticoids	Antihistamine	Antihistamine and cromoglycate	NA	Antihistamine, cromoglycate, systemic corticoids, and cyclosporine	Antihistamine	Antihistamine	Antihistamine	Antihistamine
Follow-up	5 months	3 years	6 months	1 year	6 months	NA	6 months	5 years	5 years
Complications	Mast cell leukemia	None reported	Hepatosplenomegaly	NA	Shock	NA	None reported	None reported	None reported
Vital status at follow-up (alive or dead)	Dead	Alive	Alive	Alive	Dead	Alive	Alive	Alive	Alive

	Heide et al [5] 2009			Koga et al [24] 2011	Lange et al [25] 2011		Ghiasi et al [26] 2011	Park et al [27] 2014	Folch et al [28] 2016	Chaudhary et al [6] 2019	Jenkinson et al [7] 2019	Turnbull et al [29] 2020
Number of cases	3			1	2		1	1	1	1	1	1
Sex (Male/Female)	M	M	M	M	M	F	F	F	F	M	M	F
Darier sign (positive, negative or no information)	Positive	Positive	NA	Positive	Positive	Positive	Positive	Positive	NA	Positive	Positive	NA
Systemic involvement (SI)	Yes at follow-up	Yes at follow-up	NA	No	NA	NA	NA	No	NA	No	No	No
Positive (elevated) blood and/or urine markers	Yes - urine N-methylhistamine then after 3 years of follow-up serum tryptase	Yes - urine N-methylhistamine then after 10 years of follow-up serum tryptase	Yes - serum tryptase	Yes - serum tryptase, and histamine	Yes - serum tryptase	Yes - serum tryptase	Yes - total serum tryptase	No	Yes - serum tryptase	Yes - serum tryptase	Yes - serum tryptase	Yes - serum tryptase
Bone-marrow biopsy (BMB)	NA	NA	NA	No	No	No	Yes - no involvement	No	No	Yes - no involvement	No	No
Biopsy	Yes - skin	Yes - skin	Yes - skin	Yes - skin	Yes - skin	Yes - skin	Yes - skin	Yes - skin	Yes - skin	Yes - skin	No	Yes - skin
Special stains	No	No	No	Toluidine Blue	No	No	Giemsa	No	NA	Toluidine Blue	-	Giemsa
Ultrastructural analysis (UA)	No	No		No	No	No	No	No	NA	No	-	No
IHC	Tryptase - diffuse positivity	Tryptase - diffuse positivity	Tryptase - diffuse positivity	No	CD117 (c-kit) - diffuse positivity	CD117 (c-kit) - diffuse positivity	No	CD117 (c-kit) - diffuse positivity	NA	NA	-	CD117 (c-kit) positive
Variant testing	*KIT* Asp816Val	Yes - negative, with one marker tested	No	Yes - negative	No	No	No	No	NA	*KIT* c.2447A>T (Asp816Val)	*KIT dup A502Y503*	Yes - negative, with one marker tested
Treatment	Antihistamine and cromoglycate	NA	NA	Antihistamine and cromoglycate	Antihistamine, cromoglycate, and systemic corticoids	Antihistamine, cromoglycate, and systemic corticoids	Antihistamine	Antihistamine, cromoglycate, and systemic corticoids	Antihistamine and cromoglycate	Antihistamine, cromoglycate, systemic corticoids, vincristine, dasatinib, and cladribine	Antihistamine	Antihistamine, cromoglycate, and short course of prednisolone
Follow-up	21 years	17 years	6 years	14 months	8 years	3 months	1 month	12 months	NA	7 weeks	8 months	12 months
Complications	Indolent systemic mastocytosis	Systemic mastocytosis	None reported	None reported	Hepatomegaly	None reported	Hepatosplenomegaly	None reported	Exacerbations	Hepatosplenomegaly	None reported	None reported
Vital status at follow-up (alive or dead)	Alive	Alive	Alive	Alive	Alive	Alive	Dead (cause unknown)	Alive	Alive	Dead	Alive	Alive

NA - not available   Minus (-) no information was found
